# Hydroxycarboxylic Acid Receptor 2 Is a Zika Virus Restriction Factor That Can Be Induced by Zika Virus Infection Through the IRE1-XBP1 Pathway

**DOI:** 10.3389/fcimb.2019.00480

**Published:** 2020-01-22

**Authors:** Xiaocao Ma, Xin Luo, Shili Zhou, Yanxia Huang, Cancan Chen, Changbai Huang, Li Shen, Ping Zhang, Chao Liu

**Affiliations:** ^1^Department of Immunology, Zhongshan School of Medicine, Sun Yat-sen University, Guangzhou, China; ^2^Key Laboratory of Tropical Disease Control, Ministry of Education, Zhongshan School of Medicine, Sun Yat-sen University, Guangzhou, China; ^3^Department of Pathology, First Affiliated Hospital of Sun Yat-sen University, Guangzhou, China

**Keywords:** ZIKV, HCAR2, IRE1-XBP1, CRISPR/Cas9, restriction factor, viral replication

## Abstract

Zika virus (ZIKV) is an emerging arthropod-borne virus and belongs to the *Flaviviridae* family. The infection of ZIKV has become the global health crisis because of its rapid spread and association with severe neurological disorders, including congenital microcephaly and Guillain-Barre Syndrome. To identify host factors contributing to ZIKV pathogenesis, transcriptomic landscape in ZIKV-infected cells was examined with mRNA microarray analysis and we observed that the expression of hydroxycarboxylic acid receptor 2 (HCAR2) could be significantly induced by ZIKV infection. By utilizing two IRE1 inhibitors and *XBP1*-specific shRNAs, we revealed that the up-regulation of HCAR2 expression induced by ZIKV was dependent on the IRE1-XBP1 pathway. Through the CRISPR/Cas9 system, we generated HCAR2-deficient cell clones in two cell types (human lung carcinoma epithelial A549 cell and human hepatoma Huh7.5 cell). We found that the depletion of HCAR2 significantly increased the replication level of ZIKV, including RNA levels, protein expression levels, and viral titers. In addition, our data demonstrated that the antiviral effect of HCAR2 was not involved in viral entry process and was not dependent on its antilipolytic effect on nicotinic acid/HCAR2-mediated signaling pathway. Taken together, our results indicated that HCAR2 could function as a restriction factor in control of ZIKV replication, potentially providing a novel molecular target for anti-ZIKV therapeutics.

## Introduction

Zika virus (ZIKV) is an emerging arthropod-borne virus of the *Flaviviridae* family, which includes Dengue virus (DENV), Japanese Encephalitis Virus (JEV), West Nile virus (WNV), and yellow fever (YFV). Although the infection of ZIKV in humans often leads to the asymptomatic or self-limiting disease, it also causes severe neurological disorders, such as Guillain-Barre Syndrome in adults and microcephaly in fetuses (Lazear and Diamond, 2016; Rasmussen et al., 2016). In addition to the transmission by arthropods, ZIKV can also be transmitted by other routes, including blood, sexual or vertical routes (D'Ortenzio et al., 2016; Lazear and Diamond, 2016; Rasmussen et al., 2016). As a consequence, ZIKV infection is a matter of public health emergency of international concern (Saiz et al., 2016). However, specific vaccines and drugs for ZIKV infection have not been available in the clinic.

Exploring ZIKV-related host factors is helpful for understanding the pathogenesis of ZIKV. Recently, several host genes involved in ZIKV infection have been identified. Interferon-inducible transmembrane proteins (IFITMs) were determined to inhibit ZIKV infection in the early stage of viral life cycle (Savidis et al., 2016a). Van der Hoek et al. reported that *Viperin*, one of the interferon stimulated genes, functions as a restriction factor in control of ZIKV infection (Van der Hoek et al., 2017). Furthermore, through screens of orthologous functional genomic using RNAi and CRISPR/Cas9 approaches, multiple host factors have been identified to be involved in the life cycle of ZIKV, including entry factor [AXL receptor tyrosine kinase (AXL)], endocytosis-related factors [RAB5C, member RAS oncogene family (RAB5C) and RAB guanine nucleotide exchange factor (RABGEF)], heparin sulfation-related factors [N-Deacetylase and N-Sulfotransferase 1 (NDST1), exostosin glycosyltransferase 1 (EXT1), and heparan sulfate], and the endoplasmic reticulum membrane complex (EMC) (Savidis et al., 2016b; Meertens et al., 2017; Gao et al., 2019). Nonetheless, many other ZIKV-related host factors remain to be determined.

In order to identify more novel host factors, we screened ZIKV-related genes through mRNA microarray and found that the expression of hydroxycarboxylic acid receptor 2 (HCAR2) could be dramatically induced by ZIKV infection. HCAR2, also known as GPR109A or HM74A, is a G protein-coupled receptor identified in 2003 (Soga et al., 2003; Tunaru et al., 2003; Wise et al., 2003). The HCAR2 protein has seven transmembrane structures and localizes specifically to the plasma membrane. HCAR2 is expressed in a number of cell types, including hepatocytes, immune cells, and epithelial cells (Schaub et al., 2001; Soga et al., 2003; Tunaru et al., 2003, 2005; Wise et al., 2003). The expression of this protein can be up-regulated by IFN-γ in macrophages (Schaub et al., 2001). Moreover, lipopolysaccharide (LPS), TNF-α, interleukin (IL) 1, and Toll-like receptor (TLR)-activating stimuli have been reported to induce the expression of HCAR2 in adipocytes (Digby et al., 2010; Wanders et al., 2012; Zandi-Nejad et al., 2013; Feingold et al., 2014). It has high affinity to nicotinic acid (NA) and mediates the antilipolytic effect of NA in adipocytes. Previous studies demonstrated that NA binds to HCAR2 and then decreases the level of plasma free fatty acid (FFA) or triglyceride (TG) through G (i) protein-mediated inhibition of adenylyl cyclase (Soga et al., 2003; Tunaru et al., 2003; Wise et al., 2003). In addition to the antilipolytic effects of HCAR2, the activation of this receptor also plays a role in NA/HCAR2-mediated anti-inflammatory effect (Digby et al., 2010; Montecucco et al., 2010; Lukasova et al., 2011).

In this study, we illustrated the mechanism of up-regulation of HCAR2 expression induced by ZIKV, established HCAR2-knockout (KO) cells through the CRISPR/Cas9 system, and detected the effect of HCAR2 on ZIKV replication. Our results demonstrated that ZIKV enhanced HCAR2 expression through the IRE1-XBP1 pathway and HCAR2 could reduce the replication of ZIKV. In addition, we found that the antiviral effect of HCAR2 was independent of NA/HCAR2-mediated signaling pathway.

## Result

### The Expression of HCAR2 Was Induced by Zika Virus Infection

**Figure 1:**
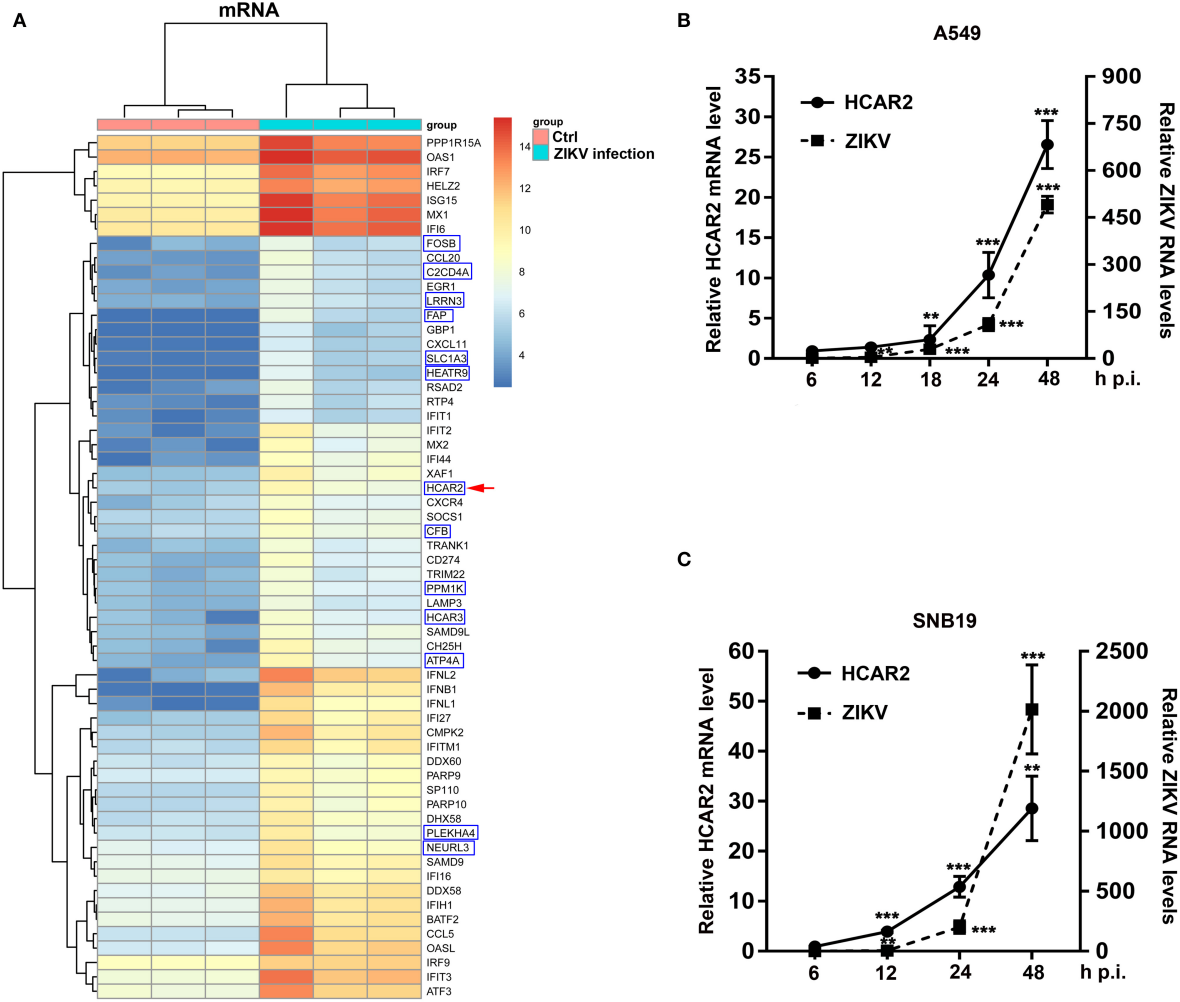
The expression of HCAR2 was induced by ZIKV infection. **(A)** The results of mRNA microarray assays in ZIKV-infected cells and control cells. A549 cells were infected with ZIKV (MOI 8) for 1 h. Then, cells were harvested at 24 h p.i. and total RNAs were extracted for mRNA microarray detection. The top 60 genes up-regulated by ZIKV infection in A549 cells were shown: the genes in the blue box were not related to type I IFN; the other genes were known to be regulated by type I IFN. *HCAR2* gene was indicated by red arrowhead. **(B)** Confirmation of *HCAR2* mRNA level in ZIKV-infected A549 cells. **(C)** Confirmation of *HCAR2* mRNA level in ZIKV-infected SNB19 cells. **(B,C)** Cells were infected with ZIKV (MOI 3) for 1 h. Then, cells were collected at indicated time points for real-time RT-PCR to measure *HCAR2* mRNA level and ZIKV RNA levels. Primers specific to *HACR2* mRNA and ZIKV *NS1* mRNA were used. Human *GAPDH* mRNA level was measured as an internal control. All the data were shown as means ± S.D. (error bars) from at least three independent experiments. Statistically significant, **p ≤ 0.01 (*t*-test); ***p ≤ 0.001 (*t*-test).

To examine changes in the transcription profile of host genes induced by ZIKV infection, human alveolar epithelial A549 cells (sensitive to ZIKV infection) were mock-infected or infected with ZIKV at an MOI of 8 and harvested at 24 h post-infection (p.i.). Then, total RNAs were extracted for mRNA microarray to evaluate the regulation of host genes in ZIKV-infected A549 cells. In the microarray results, ZIKV infection up-regulated 139 genes and down-regulated 1 gene (criteria: *p* < 0.05, fold change>2). Top 60 genes (up to 5-fold change) were selected from the 139 up-regulated genes (Figure 1A). We found that most genes (47 genes) were known to be regulated by type I interferon (IFN) and 13 genes were not related to type I IFN. Among these genes that are not related to type I IFN, *HCAR2* and *HCAR3* belong to G protein-coupled receptors that are involved in the metabolic effects of nicotinic acid (Zellner et al., 2005). We were especially interested in HCAR2, as its mRNA level was enhanced by approximately 11-fold at 24 h p.i. (Figure 1A).

To verify the accuracy of the mRNA microarray analysis, we infected A549 cells with ZIKV at an MOI of 3 and harvested the cells at different time points (6, 12, 18, 24, and 48 h). Subsequently, total RNAs were extracted for quantitative real-time PCR (qRT-PCR) to detect *HCAR2* mRNA level and ZIKV RNA levels. As shown in Figure 1B, ZIKV infection up-regulated the expression of HCAR2 in a time-dependent manner and the highest fold change was around 27 at 48 h p.i. As ZIKV infection is associated with severe neurological diseases (Pierson and Diamond, 2018), we detected the relationship between HCAR2 and ZIKV in human glioblastoma SNB19 cells. Similarly, the expression of HCAR2 in SNB19 cells was also increased in response to ZIKV infection in a time-dependent manner (~5-fold at 12 h p.i., ~13-fold at 24 h p.i., and ~29-fold at 48 h p.i.) (Figure 1C). Moreover, up-regulation of HCAR2 in SNB19 cells was earlier than that in A549 cells. This result suggested that the induction of HCAR2 by ZIKV infection was not cell-specific.

### The Up-Regulation of HCAR2 Expression Induced by Zika Virus Infection Was Dependent on the IRE1-XBP1 Pathway

**Figure 2:**
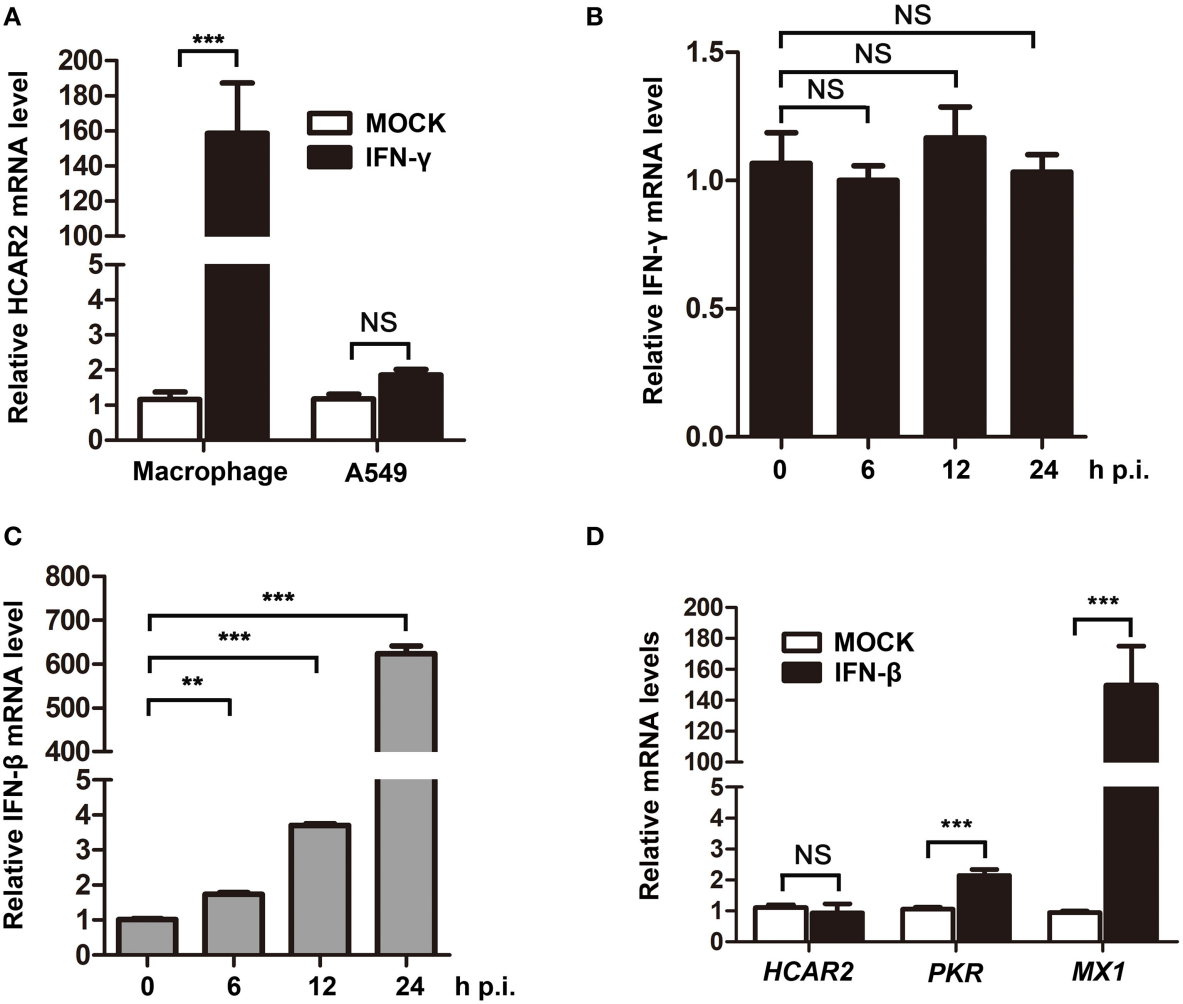
The up-regulation of HCAR2 expression induced by ZIKV infection was independent of IFNs. **(A)** Impact of IFN-γ on HCAR2 expression. After treatment with 100 nM of PMA for 24 h, THP-1 cells were differentiated into macrophages. A549 cells or macrophages were treated with 100 ng/ml of IFN-γ for 24 h and harvested for total RNAs extraction. Then, real-time RT-PCR assay was performed to detect *HCAR2* mRNA level. **(B)** Effect of ZIKV infection on IFN-γ expression. **(C)** Effect of ZIKV infection on IFN-β expression. **(B,C)** A549 cells were infected with ZIKV (MOI 3) for 1 h. Then, total RNAs extracted from these infected cells at indicated time points (0, 6, 12, and 24 h p.i.) were detected by real-time RT-PCR assay to measure *IFN-*γ **(B)** or *IFN-*β **(C)** mRNA level. **(D)** Impact of IFN-β on HCAR2 expression. A549 cells were treated with 1000 unit/ml of IFN-β for 24 h and harvested for total RNAs extraction. Real-time RT-PCR assay was performed to detect *HCAR2, PKR*, or *MX1* mRNA level. **(A–D)** Human *GAPDH* mRNA level was measured as an internal control. All the data were shown as means ± S.D. (error bars) from at least three independent experiments. NS, not significant; statistically significant, **p ≤ 0.01 (*t*-test); ***p ≤ 0.001 (*t*-test).

As previous studies reported that the expression of HCAR2 could be induced by IFN-γ in macrophages and ZIKV was demonstrated to activate type II IFN signaling (Wanders et al., 2012; Zandi-Nejad et al., 2013; Chaudhary et al., 2017), we speculated that ZIKV might up-regulate HCAR2 expression by stimulating secretion of IFN-γ. To test this hypothesis, we firstly detected the impact of IFN-γ on HCAR2 expression in A549 cells. In this experiment, THP-1-differentiated macrophages were used as the positive control. Macrophages and A549 cells were treated with IFN-γ for 24 h and then harvested for qRT-PCR to measure *HCAR2* mRNA level. We found that *HCAR2* mRNA level could not be induced by IFN-γ treatment in A549 cells, but could be significantly enhanced by IFN-γ treatment in macrophages (~160-fold) (Figure 2A). Then, the expression of IFN-γ in ZIKV-infected A549 cells was also examined. The cells were infected with ZIKV (MOI 3) and harvested at 24 h p.i. for qRT-PCR to measure *IFN-*γ mRNA level. However, IFN-γ expression was not stimulated by ZIKV in A549 cells (Figure 2B). Considering that ZIKV can significantly stimulate the expression of IFN-β and many host genes are induced by IFN-β (Frumence et al., 2016; Lee et al., 2018), the expression of IFN-β in ZIKV-infected A549 cells and the effect of IFN-β on *HCAR2* mRNA level were also detected. Although ZIKV could significantly stimulate IFN-β expression from 12 to 24 h p.i. in A549 cells (Figure 2C), the expression of HCAR2 was not enhanced by IFN-β treatment (Figure 2D). In contrast, the expression of control IFN-stimulated genes [protein kinase R (PKR) and MX dynamin like GTPase 1 (MX1)] could be up-regulated by type I IFN (Figure 2D). These data demonstrated that the increased expression of HCAR2 induced by ZIKV infection was independent of type I and II IFNs.

**Figure 3:**
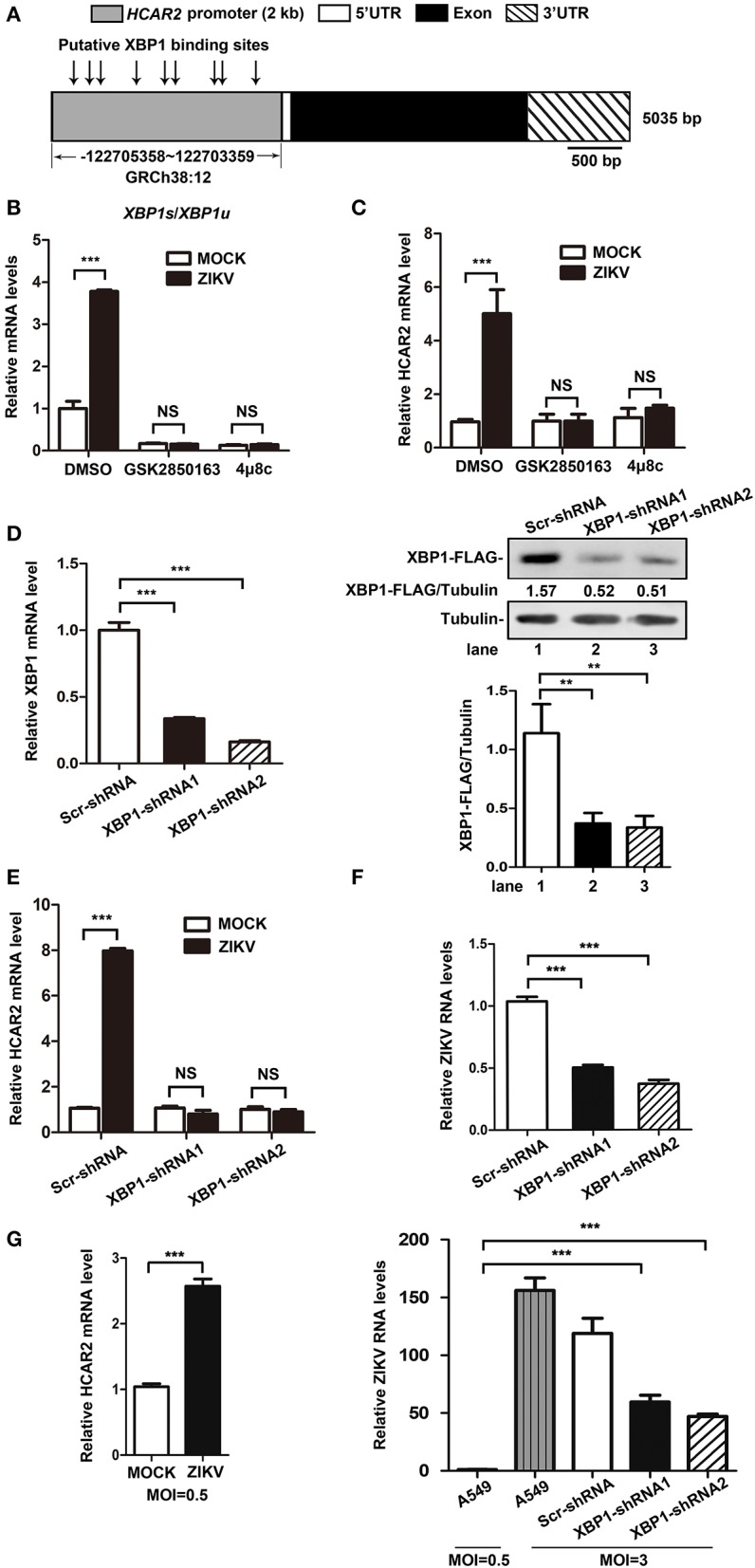
The increased expression of HCAR2 induced by ZIKV infection was dependent on the IRE1-XBP1 pathway. **(A)** Putative XBP1 binding sites on the promoter region of *HCAR2*. **(B)** Effect of IRE1 inhibitors on XBP1 splicing. A549 cells were treated with 50 μM of two IRE1 inhibitors (GSK2850163 and 4μ8c) for 24 h and then total RNAs were extracted. Real-time RT-PCR assay was performed to detect *XPB1u* and *XBP1s* mRNA level. **(C)** Effect of IRE1 inhibitors on HCAR2 expression in the context of ZIKV infection. A549 cells were infected with ZIKV for 1 h at an MOI of 3 and then treated with 50 μM of two IRE1 inhibitors (GSK2850163 and 4μ8c), respectively. Cells were collected at 24 h p.i. and total RNAs were extracted for real-time RT-PCR assay to measure *HCAR2* mRNA level. **(D)** Confirmation of XBP1 knockdown efficiency by real-time RT-PCR and western blot assays. A549 cells were infected with pLKO.1-*XBP1*-shRNA or pLKO.1-Scr-shRNA lentivirus for 1 h and then selected with puromycin for 1 week. Total RNAs were extracted for real-time RT-PCR assay to measure *XBP1* mRNA level. pLKO.1-*XBP1*-shRNA or pLKO.1-Scr-shRNA lentivirus infected A549 cells were selected with puromycin for 1 week and then transfected with 0.8 μg of XBP1-FLAG-expressing plasmid (CSII-EF-MCS-IRES2-Venus-XBP1-FLAG). Cells were harvested at 24 h post-transfection for western blot assay to measure *XBP1*-shRNAs knockdown efficiency. **(E)** Effect of XBP1 knockdown on HCAR2 expression. **(F)** Effect of XBP1 knockdown on ZIKV RNA levels. **(E,F)** A549 XBP1-knockdown and control cells were infected with ZIKV (MOI 3) for 1 h and harvested at 24 h p.i. Total RNAs were extracted for real-time RT-PCR assay to measure *HCAR2* mRNA level **(E)** and ZIKV RNA levels **(F)**. **(G)** Effect of a lower MOI of ZIKV infection on HCAR2 expression (left panel). Comparison of viral RNA levels (right panel). A549 and A549 XBP1-knockdown cells were infected with ZIKV at different MOIs (MOI 0.5 or MOI 3 for A549 cells, MOI 3 for XBP1-knockdown cells). Cells were collected at 24 h p.i. for real-time RT-PCR assay to measure *HCAR2* mRNA level and ZIKV RNA levels. **(B–G)** Human *GAPDH* mRNA level was measured as an internal control. All the data were shown as means ± S.D. (error bars) from at least three independent experiments. Statistically significant, **p ≤ 0.01 (*t*-test); ***p ≤ 0.001 (*t*-test); NS, not significant.

To elucidate the mechanism of the up-regulation of HCAR2 expression induced by ZIKV infection, we analyzed the transcription factor binding sites on the promoter region of HCAR2 with the online bioinformatics software (http://alggen.lsi.upc.es/cgi-bin/promo_v3/promo/promoinit.cgi?dirDB=TF_8.3). Interestingly, multiple putative X-box binding protein 1 (XBP1) binding sites were predicted on *HCAR2* promoter region (Figure 3A), implying a potential association between HCAR2 and XBP1. XBP1, an important transcription factor, is a substrate of phosphorylated inositol-requiring enzyme 1 (IRE1). IRE1-mediated XBP1 splicing is an important branch of unfolded protein response (UPR) (Ron and Walter, 2007). Previous studies have reported that in response to ZIKV infection, phosphorylated IRE1 splices a 26-nucleotide intron from full-length *XBP1* mRNA (*XBP1u*), resulting in the splicing *XBP1* form (*XBP1s*). *XBP1s* encodes spliced XBP1 (XBP1s), which is a transcription factor up-regulating expression of some genes involved in endoplasmic reticulum (ER) chaperones and ER-association degradation proteins (Tan et al., 2018). Thus, we hypothesized that ZIKV might induce HCAR2 expression through activating the IRE1-XBP1 pathway. To verify this hypothesis, two kinds of IRE1 inhibitors (GSK2850163 and 4μ8c) were used in the context of ZIKV infection (MOI 3) to detect their effects on the expression of HCAR2 by qRT-PCR assay. As shown in the Figure 3B, GSK2850163 (an inhibitor for IRE1 kinase activity) (Concha et al., 2015) and 4μ8c (an inhibitor for IRE1 endoribonulease activity) (Cross et al., 2012) dramatically inhibited the splicing of *XBP1* in both mock and infected cells. Moreover, the mRNA level of HCAR2 was not increased by ZIKV infection with GSK2850163 or 4μ8c treatment (Figure 3C). To further confirm the role of XBP1 in the induction of HCAR2, the XBP1-knockdown (KD) cells were constructed by the infection of *XBP1*-specific shRNA-expressing lentivirus and its knockdown efficiency was shown in Figure 3D. The cells were then infected with ZIKV at an MOI of 3 and harvested at 24 h p.i. for qRT-PCR assay to examine *HCAR2* mRNA level. Similarly, the mRNA level of *HCAR2* in XBP1-KD cells was not enhanced in response to ZIKV infection (Figure 3E). To exclude the possibility that the lower level of HCAR2 in XBP1-KD cells is due to less replication of ZIKV, we compared viral RNA levels in the control and XBP1-KD cells (MOI 3). The qRT-PCR data showed that the ZIKV RNA levels were reduced by XBP1 knockdown (~1-fold at 24 h p.i.) (Figure 3F). Furthermore, the expression level of HCAR2 in response to a lower MOI of ZIKV infection (MOI 0.5) was also examined. The result showed that even at an MOI of 0.5, *HCAR2* mRNA level was also enhanced by viral infection (~2.6-fold at 24 h p.i.) (Figure 3G, left panel). In addition, viral RNA levels in ZIKV-infected XBP1-KD cells (MOI 3) and A549 cells (MOI 0.5) were compared. As shown in the Figure 3G (right panel), the ZIKV RNA levels in XBP1-KD cells (MOI 3) (~65-fold at 24 h p.i.) were much higher than that in normal A549 cells (MOI 0.5). Therefore, these data indicated that ZIKV up-regulated HCAR2 expression through the IRE1-XBP1 pathway.

### The Replication of Zika Virus Was Up-Regulated in HCAR2-KO Cells

**Figure 4:**
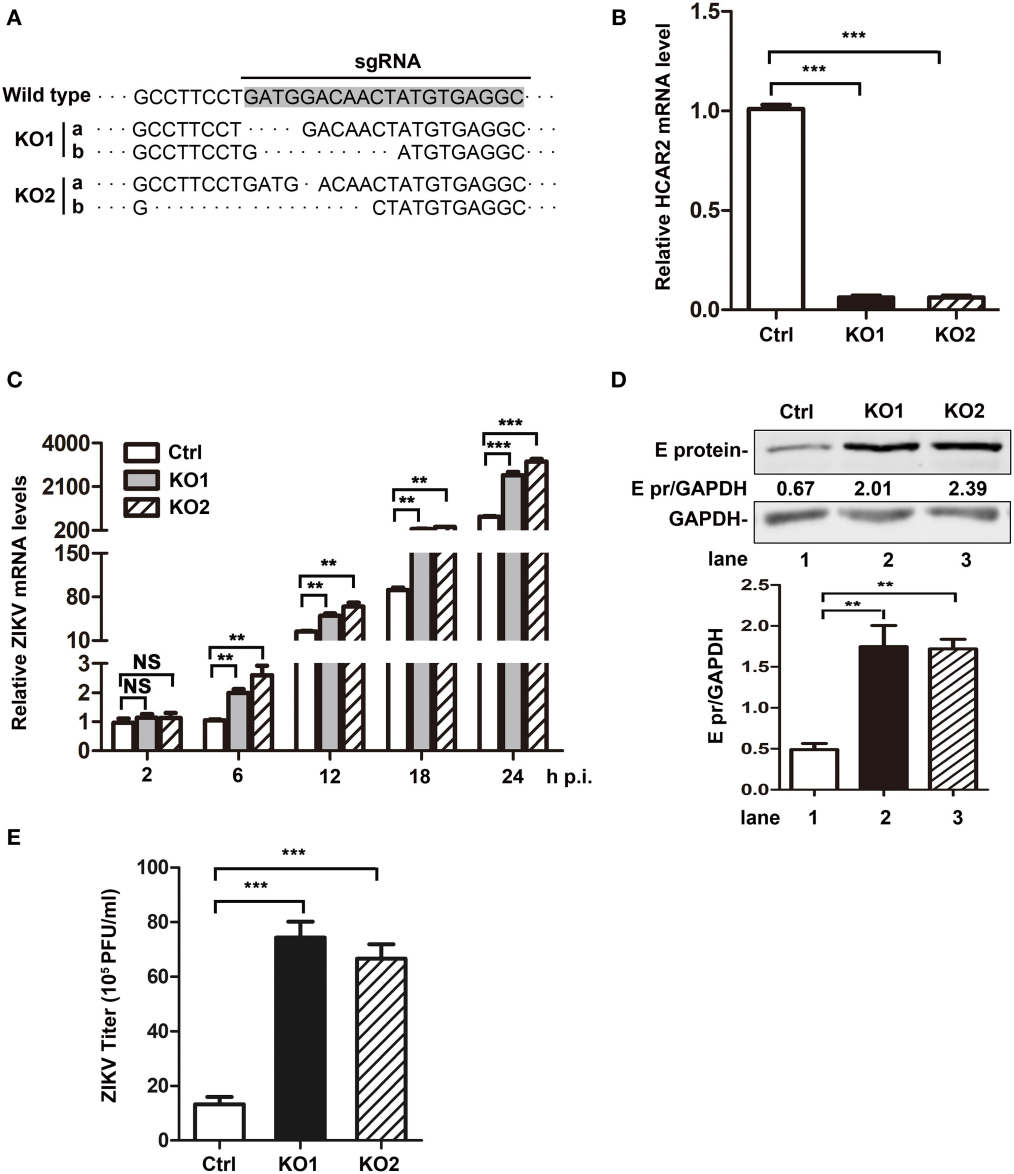
HCAR2 knockout enhanced the replication of ZIKV in A549 cells. **(A)** Confirmation of HCAR2 knockout efficiency by DNA sequencing. **(B)** Confirmation of HCAR2 knockout efficiency by real-time RT-PCR assay. **(A,B)** The CRISPR/Cas9 mediated A549 HCAR2-KO1 or A549 HCAR2-KO2 cell clone was isolated. Then, the genomic DNAs or total RNAs were extracted for DNA sequencing **(A)** or real-time RT-PCR assay **(B)** with *HCAR2*-specific probes. **(C–E)** The effect of HCAR2 depletion on ZIKV RNA level **(C)**, E protein level **(D)**, and titer **(E)**. A549 HCAR2-deficient cells were infected with ZIKV (MOI 3) for 1 h and collected at 2, 6, 12, 18, and 24 h p.i. The viral RNA level was measured by real-time RT-PCR using primers specific to ZIKV *NS1* mRNA. Human *GAPDH* mRNA level was measured as an internal control **(C)**. Western blotting was performed to detect ZIKV E protein expression at 24 h p.i. **(D)**. GAPDH served as an internal control. The supernatants of infected cells were harvested at 24 h p.i. and plaque assay was performed to measure viral titer **(E)**. All the data were shown as means ± S.D. (error bars) from at least three independent experiments. Statistically significant, **p ≤ 0.01 (*t*-test); ***p ≤ 0.001 (*t*-test); NS, not significant.

As the above results showed a close relationship between HCAR2 and ZIKV, we were interested in the effect of HCAR2 on ZIKV replication. Firstly, A549 HCAR2-KO cells were generated with the CRISPR/Cas9 system and two monoclonal cell lines (HCAR2-KO1 or HCAR2-KO2) were selected. To examine the knockout efficiency, we purchased various brands of anti-HCAR2 antibody from different companies and tested the expression of HCAR2 in HCAR2-KO and control cells by western blot and flow cytometry analysis. Unfortunately, all of these antibodies did not work, which might be due to the seven transmembrane-spanning structure and cellular membrane-localization of this protein (Schaub et al., 2001; Martin et al., 2009). Instead, DNA sequencing and qRT-PCR experiments were used to test the knockout efficiency of HCAR2. As shown in Figures 4A,B, *HCAR2* gene was successfully edited in HCAR2-KO1 or HCAR2-KO2 cells. To detect whether HCAR2 affects the ZIKV replication, the viral RNA levels, protein expression, and titers from HCAR2-KO and control cells were compared through qRT-PCR, western blot, and plaque assay. A549 cells were infected with ZIKV (MOI 3) and harvested at 24 h p.i for qRT-PCR or western blot assay. Compared to the control cells, the viral RNA levels in HCAR2-KO cells were increased by ~2.5-fold at 6 h p.i., ~2.6-fold at 12 h p.i., ~3.7-fold at 18 h p.i., and ~4-fold at 24 h p.i., respectively (Figure 4C). The western blot result demonstrated that ZIKV E protein level in HCAR2-KO cells were significantly higher than the control group (~3.5-fold) (Figure 4D). Next, the supernatants of the infected cells were collected at 24 h p.i. and titered. As shown in Figure 4E, the viral yields of HCAR2-deficient cells were dramatically enhanced (~7-fold). These data suggested that HCAR2 could reduce the replication of ZIKV.

### HCAR2 Also Significantly Reduced Zika Virus Replication in Hepatocytes

**Figure 5:**
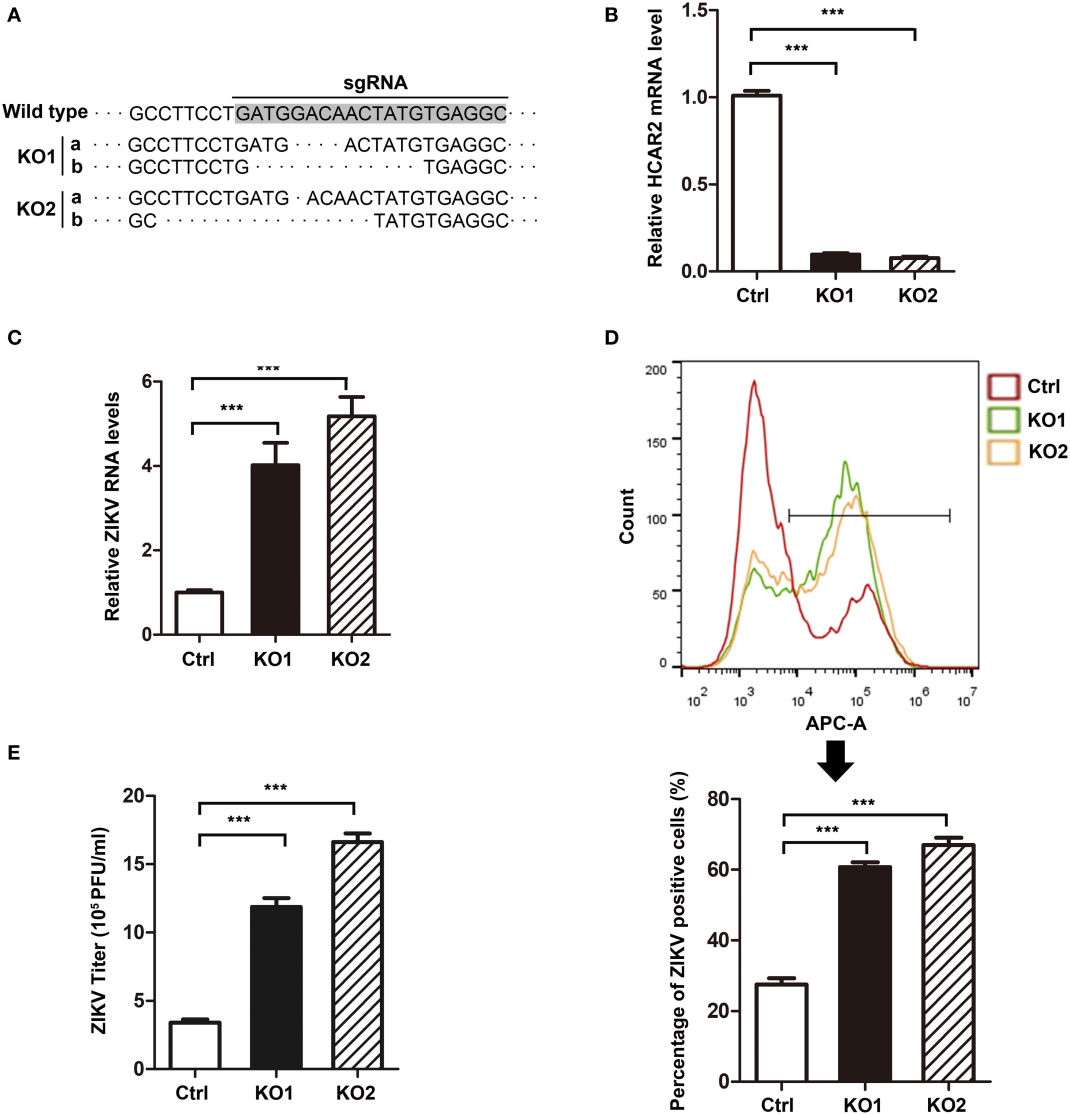
HCAR2 inhibited ZIKV replication in hepatocytes. **(A,B)** Confirmation of HCAR2 knockout efficiency by DNA sequencing **(A)** and real-time RT-PCR assay **(B)**. The CRISPR/Cas9 generated Huh7.5 HCAR2-KO1 and HCAR2-KO2 cell clones were isolated. The genomic DNAs or total RNAs were extracted, respectively for DNA sequencing **(A)** or real-time RT-PCR assay **(B)** with *HCAR2*-specific probes. **(C–E)** Effect of HCAR2 on ZIKV RNA level **(C)**, infection efficiency **(D)**, and titer **(E)**. Huh7.5 HCAR2-deficient cells were infected with ZIKV (MOI 1) for 1 h and harvested at 24 h p.i. Real-time RT-PCR assay was performed to measure viral RNA level at 24 h p.i. by using primers specific to ZIKV *NS1* mRNA. Human *GAPDH* mRNA level was measured as an internal control **(C)**. The percentages of ZIKV E protein-positive staining cells in HCAR2-KO and control group were detected by flow cytometry at 24 h p.i. **(D)**. The supernatants of the viral infected Huh7.5 cells were collected and titered by plaque assay at 24 h p.i. **(E)**. All the data were shown as means ± S.D. (error bars) from at least three independent experiments. Statistically significant, ***p ≤ 0.001 (*t*-test).

Considering that HCAR2 plays an important role in lipid metabolism and hepatocytes are the typical adipocytes (Soga et al., 2003; Tunaru et al., 2003; Wise et al., 2003), we further examined the effect of HCAR2 on ZIKV replication in hepatocytes. We generated HCAR2-KO Huh7.5 cells with CRISPR/Cas9 system and two monoclonal cell lines (HCAR2-KO1 or HCAR2-KO2) were selected. The knockout effect of HCAR2 was confirmed by DNA sequencing and qRT-PCR assay (Figures 5A,B). Next, the HCAR2-KO cells were infected with ZIKV (MOI 1) and harvested at 24 h p.i. to analyze the viral RNA levels and infection efficiency. The qRT-PCR result showed that in HCAR2-deficient hepatocytes, the viral RNA levels were enhanced by approximately 6-fold at 24 h p.i. (Figure 5C). Moreover, the percentages of ZIKV E protein-positive staining cells in HCAR2-KO group (~65%) detected by flow cytometry at 24 h p.i. were higher than that in the control group (~27%) (Figure 5D), suggesting that the infection efficiency of ZIKV was reduced by HCAR2. Furthermore, the supernatants of the infected hepatocytes were collected and titered at 24 h p.i. The plaque assay demonstrated that the viral titers of Huh7.5 HCAR2-KO cells were dramatically up-regulated (~4-fold) (Figure 5E). Altogether, these results indicated that in hepatocytes, HCAR2 could also reduce the ZIKV replication.

### The Reduction Effect of HCAR2 on Zika Virus Replication Was Not Involved in Viral Attachment or Endocytosis Process

**Figure 6:**
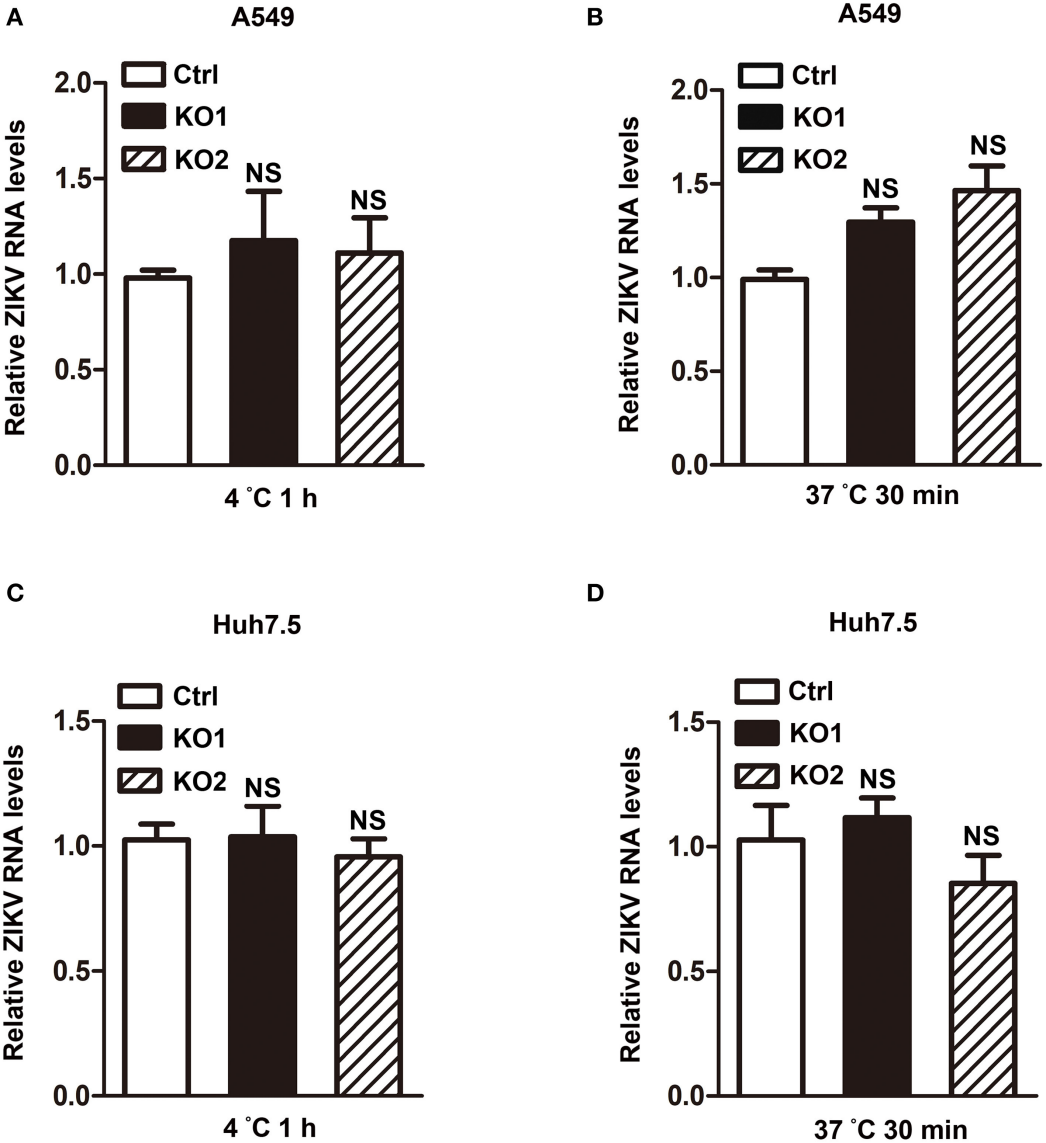
HCAR2 had no influence on the viral attachment or endocytosis step. **(A,C)** Effect of HCAR2 on viral attachment step. Cells were inoculated with ZIKV (MOI 3 for A549 cells, MOI 1 for Huh7.5 cells) at 4°C for 1 h. Cells were then washed with PBS and harvested for RNA extraction. **(B,D)** Effect of HCAR2 on ZIKV endocytosis process. Cells were inoculated with ZIKV (MOI 3 for A549 cells, MOI 1 for Huh7.5 cells) at 37°C for 1 h. Cells were then washed with glycine-HCl pH3.0 buffer and collected for RNA extraction. **(A–D)** Real-time RT-PCR assay was performed to detect viral RNA levels by using primers specific to ZIKV *NS1* mRNA. Human *GAPDH* mRNA level was measured as an internal control. All the data were shown as means ± S.D. (error bars) from at least three independent experiments. NS, not significant.

Considering that HCAR2 is a receptor and localizes to the cell membrane (Schaub et al., 2001; Soga et al., 2003; Tunaru et al., 2003; Wise et al., 2003; Martin et al., 2009), we hypothesized that HCAR2 might play an antiviral role in the process of ZIKV attachment or endocytosis. To verify this speculation, we firstly determined the effect of HCAR2 on ZIKV attachment by incubating the virions with A549 HCAR2-KO and control cells at 4°C for 1 h. The viral RNA levels in cells were detected by qRT-PCR assay to indicate the amounts of viral particles that bound to the cell membrane. The qRT-PCR result showed that the viral RNA levels of HCAR2-KO cells were similar to those in the control cells (Figure 6A), implying that HCAR2 was not involved in the viral attachment process. Then, to test its impact on the viral endocytosis stage, cells were incubated with ZIKV at 37°C for 30 min to allow for viral attachment and internalization. Total RNAs in the whole cells were extracted for qRT-PCR analysis to detect the internalized viral RNA levels. As shown in Figure 6B, the viral RNA levels were comparable in the control and HCAR2-deficient cells. And also, the same results were observed in hepatocytes (Figures 6C,D). Therefore, these data suggested that HCAR2 did not act at the viral endocytosis step.

### The Antiviral Effect of HCAR2 Was Independent of NA/HCAR2-Mediated Signaling Pathway

**Figure 7:**
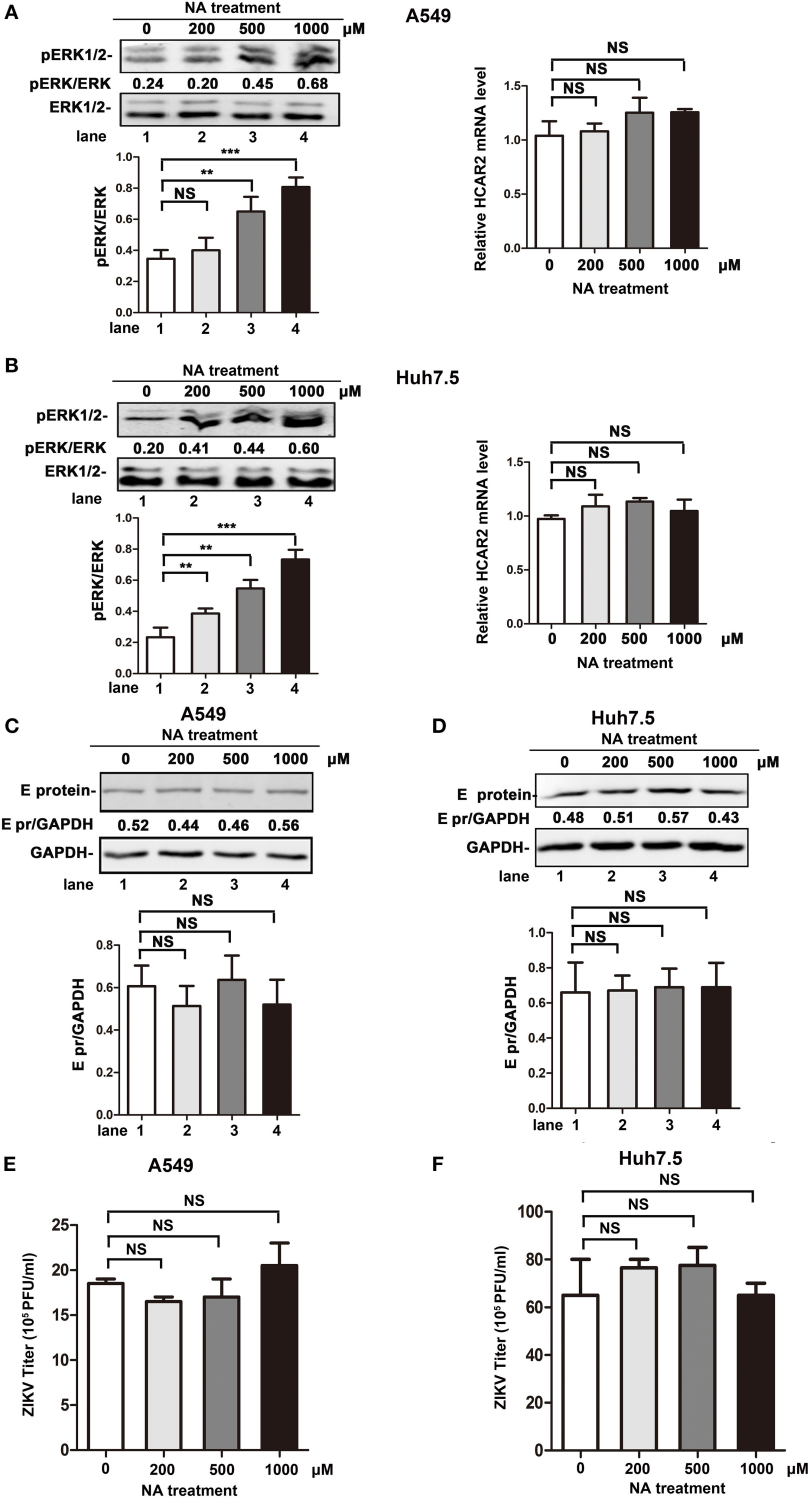
The antiviral effect of HCAR2 was independent of NA/HACR2-mediated signaling pathway. **(A,B)** Effect of NA on phosphorylation of ERK1/2 and the expression of HCAR2. Cells were treated with different amounts of NA (200, 500, 1,000 μM) and then collected for western blot and real-time RT-PCR assays. The phosphorylation of ERK1/2 was examined by western blotting (left panels). Real-time RT-PCR assay was performed to measure *HCAR2* mRNA level (right panels). **(C,D)** Impact of NA on ZIKV E protein expression level. **(E,F)** Effect of NA on ZIKV titer. **(C–F)** Cells were pre-treated with different amounts of NA (200, 500, 1,000 μM) for 1 h and then infected with ZIKV (MOI 3 for A549 cells, MOI 1 for Huh7.5 cells) for 1 h. At 24 h p.i., cells were harvested for western blotting to measure ZIKV E protein expression level **(A,B)**. GAPDH served as an internal control. The supernatants of infected cells were collected at 24 h p.i. for viral titration by plaque assay **(C,D)**. All the data were shown as means ± S.D. (error bars) from at least three independent experiments. Statistically significant, **p ≤ 0.01 (*t*-test); ***p ≤ 0.001 (*t*-test); NS, not significant.

Previous studies reported that HCAR2, as the receptor of NA, plays an important role in the lipolytic effect of NA. The activation of HCAR2 inhibits the degradation of triglyceride, leading to a decrease in plasma free fatty acid (FFA) and adenosine triphosphate (ATP) production (Soga et al., 2003; Tunaru et al., 2003; Wise et al., 2003). As lipid metabolism is important for the replication process of flaviviruses (Heaton and Randall, 2010; Martin-Acebes et al., 2016, 2019; Zhang et al., 2017; Martins et al., 2018; Osuna-Ramos et al., 2018; Pombo and Sanyal, 2018), we speculated that the reduction effect of HCAR2 on viral replication might be dependent on NA/HCAR2-mediatd lipid metabolism pathway. To explore this hypothesis, the impact of NA on ZIKV replication was examined. As previous studies reported that activation of HCAR2 by NA can induce the phosphorylation of extracellular signal-regulated kinase 1/2 (ERK1/2), we set ERK1/2 as an activation indicator of NA/HCAR2-mediated pathway (Tunaru et al., 2003; Li et al., 2010). As shown in Figures 7A,B (left panel), the enhancement of phosphorylated ERK1/2 level in A549 or Huh7.5 cells was correlated with the concentration of NA, indicating that NA/HCAR2-mediated pathway was successfully activated. Then, A549 and Huh7.5 cells were pre-treated with different concentrations of NA (200, 500, or 1,000 μM) for 1 h and then infected with ZIKV. At 24 h p.i., these cells and supernatants were collected for western bolt or plaque assay. However, compared to the control group, the expression of ZIKV E protein in NA-treatment group remained unchanged (Figures 7C,D). Similarly, the viral yields were also comparable in the control and NA-treated cells (Figures 7E,F), demonstrating that the antiviral effect of HCAR2 was independent of NA/HCAR2-signaling pathway.

## Discussion

ZIKV is an emerging arbovirus and can cause severe neurological disorders, including Guillain-Barre Syndrome and microcephaly (Saiz et al., 2016). Identifying ZIKV-related host factors contributes to the understanding of viral pathogenesis. Recently, several host genes have been reported to up-regulate or down-regulate the replication of ZIKV, for example AXL, suppressor of cytokine signaling 3 (SOCS3), indoleamine 2,3-dioxygenase 1 (IDO-1), and endoplasmic reticulum membrane complex (EMC) function as ZIKV-dependency factors, while virus-inhibitory protein, endoplasmic reticulum-associated, IFN-inducible (Viperin), fragile X mental retardation protein (FMRP), and interferon-inducible transmembrane proteins (IFITM) family members act as inhibitory factors (Savidis et al., 2016a,b; Meertens et al., 2017; Sun et al., 2017; Van der Hoek et al., 2017; Soto-Acosta et al., 2018). In this study, we identified HCAR2, a G protein-coupled receptor which expression is induced by ZIKV, functions as a restriction factor for ZIKV replication.

Firstly, the data obtained with mRNA microarray and qRT-PCR assays showed that the expression of HCAR2 was significantly induced by ZIKV infection, indicating a strong association between HCAR2 and ZIKV life cycle. In 2017, Li et al. have performed RNA-sequencing assay in ZIKV-infected (GZ01/2016 strain) A549 cells (Li et al., 2017). The top 50 genes up-regulated by ZIKV infection were shown, but *HCAR2* gene was not in the list. The differences between our microarray results and theirs may be due to the different methods used in the experiments.

Until now, several factors have been reported to regulate the expression of HCAR2, including TNF-α, LPS, Interleukin 1, Zymosan, lipoteichoic acid, polyinosine-polycytidylic acid, and other Toll-like receptor activators (Digby et al., 2010; Wanders et al., 2012; Zandi-Nejad et al., 2013; Feingold et al., 2014). Nevertheless, the mechanism of regulating HCAR2 expression is still unclear. After ruling out the involvement of type I and II IFNs in its induction by ZIKV, we turned our attention to *HCAR2* promoter region. Interestingly, we predicted multiple putative XBP1 binding sites on *HCAR2* promoter region and experimentally demonstrated that XBP1 is important for the up-regulation of HCAR2 induced by ZIKV. XBP1 is a substrate of IRE1, and transcriptionally up-regulates the expression of some genes involved in ER chaperones and ER-association degradation (ERAD) proteins (Tan et al., 2018). Our study compared the *HCAR2* mRNA levels in the context of ZIKV infection with treatment of IRE1 inhibitors or *XBP1*-specific shRNAs. Interestingly, the result suggested that ZIKV up-regulated the HCAR2 expression through the IRE1-XBP1 pathway. Infection by viruses within the *Flaviviridae* family disrupts the normal ER function and then induces ER stress. IRE1-XBP1 pathway is an arm of UPR which is activated by ER stress. It plays an important role in the ER-tropic viral infection (Yu et al., 2006; Ambrose and Mackenzie, 2011; Pena and Harris, 2011; Saeed et al., 2011). However, different viruses trigger different cell responses mediated by IRE1-XBP1. JEV infection activates the IRE1-XBP1 pathway and benefits for its infectivity by activating the regulated IRE1-dependent decay pathway (Yu et al., 2006). In contrast, HCV-activated IRE1-XBP1 pathway results in the increased expression of ER degradation-enhancing α-mannosidase-like protein 1 (EDEM1) and EDEM3 that in turn accelerate HCV glycoprotein E2 degradation, leading to a reduction of virus production (Saeed et al., 2011). Previous study also reported that ZIKV infection significantly activated the IRE1-XBP1 pathway to regulate cellular apoptosis mediated by CHOP (Tan et al., 2018). In our study, we demonstrated that the IRE1-XBP1 pathway mediates the induction of HCAR2 upon ZIKV infection. The cellular responses to the up-regulation of HCAR2 induced by ZIKV through IRE1-XBP1 pathway need further study.

To detect whether HCAR2 is involved in the replication of ZIKV, we generated two types of HCAR2-deficient cells (A549 HCAR2-KO cell and Huh7.5 HCAR2-KO cell). Depletion of HCAR2 resulted in significant increase of ZIKV RNA levels, viral E protein level, and viral yields, indicating that HCAR2 is a restriction factor for ZIKV replication. To explore the antiviral mechanism of HCAR2, we have detected its association with the known possibilities. As HCAR2 localizes specifically to the plasma membrane and acts as a G protein-coupled receptor for NA to mediate its lipolytic effect (Schaub et al., 2001; Soga et al., 2003; Tunaru et al., 2003, 2005; Wise et al., 2003), we tested the possibility that HCAR2 functions in viral entry process, and our results indicated that HCAR2 was not involved in ZIKV attachment or endocytosis process. Of note, since the functions of HCAR2 are mostly associated with NA/HCAR2-mediated signaling pathway (Schaub et al., 2001; Soga et al., 2003; Tunaru et al., 2003, 2005; Wise et al., 2003; Montecucco et al., 2010; Lukasova et al., 2011), we further examined whether the antiviral function of HCAR2 depends on this pathway by NA treatment. Unfortunately, our data demonstrated that the reduction effect of HCAR2 on ZIKV was independent of NA/HCAR2-mediated signaling pathway. Based on these observations, we ruled out its involvement in the known regulatory axis. Considering that HCAR2 significantly reduced both RNA level and E protein level of ZIKV, we speculated that it might play a role in the stage of ZIKV RNA synthesis or viral protein translation. Therefore, a ZIKV replicon system is needed to further distinguish between its impact on viral RNA replication and protein translation. Moreover, if the antibody against HCAR2 works, future studies (such as the co-localization or co-precipitation of HCAR2 with viral RNA in ZIKV-infected cells and the interaction of HCAR2 with ZIKV replication complex proteins) will be helpful for determining a possible interaction of HCAR2 at sites of ZIKV replication.

Taken together, our work provides the first evidence to show a relationship between host HCAR2 and virus: the expression of HCAR2 could be significantly up-regulated by ZIKV infection through the IRE1-XBP1 pathway, and in turn, HCAR2 reduces the ZIKV replication. Moreover, the antiviral role of HCAR2 in ZIKV replication was not involved in viral entry step and independent of NA/HCAR2-mediated signaling pathway. Our study identified a novel restriction factor for ZIKV replication. Further investigations to reveal the mechanism of how HCAR2 restricts viral replication will benefit the understanding of ZIKV pathogenesis and potentially provide a novel molecular target for anti-ZIKV therapeutics.

## Materials and Methods

### Cell Lines

Human lung carcinoma epithelial cells (A549), human glioblastoma cells (SNB19), and human hepatoma cells (Huh7.5) were maintained in Dulbecco′s modified Eagle′s medium (DMEM, Gibco) supplemented with 10% fetal bovine serum (FBS) (Gibco), 100 units/ml of penicillin and streptomycin (Invitrogen) at 37°C with 5% CO_2_. Human monocytic leukemia cells (THP-1) were cultured in RPMI 1640 conditioned medium (Gibco) supplemented with 10% FBS, 100 units/ml of penicillin and streptomycin (Invitrogen) at 37°C with 5% CO_2_.

### Virus and Virus Titration

The ZIKV (H/PF/2013 strain) was provided by Guangzhou Centers for Disease Control. Virus was propagated in Vero cells. Viral stocks were stored at −80°C. Virus titer was detected by standard plaque or focus-forming assay (FFA) in Vero cells as previously described (Wang et al., 2019). Visible plaques were counted at 3–4 days post-infection.

### mRNA Microarray Accession Number

mRNA microarray data have been deposited to the NCBI GEO database (https://www.ncbi.nlm.nih.gov/geo/query/acc.cgi?acc=GSE124094). The accession number is GSE124094.

### Quantitative Real-Time PCR (qRT-PCR)

Total RNAs were extracted using Trizol reagent (Invitrogen) and reverse transcribed using M-MLV reverse transcriptase (Promega) according to the manufacturer's instructions. Quantitative real-time PCR analysis was preformed (SYBR Premix ExTaq, TaKaRa) on a CFX96 Real-Time System (Bio-Rad). The following primers were used in this report: *HCAR2*-F: 5′-ACTATGTGAGGCGTTGGGAC-3′, *HCAR2*-R: 5′-GCTGTCCGATTGGAGATCT-3′; ZIKV *NS1*-F: 5′-GTCAGAGCAGCAAAGACAA-3′, ZIKV *NS1*-R: 5′-CAGCCTCCTTTCCCTTAACA-3′; *IFN-*β-F: 5′-AAACTCATGAGCAGTCTGCA-3′, *IFN-*β-R: 5′-AGGAGATCTTCAGTTTCGGAGG-3′; *IFN-*γ-F: 5′-TGGAGACCATCAAGGAAGACA-3′, *IFN-*γ-R: 5′-GTTCAGCCATCACTTGGATGA-3′; *PKR*-F: 5′-AGCAGTTCTTCCATCTGACTC-3′, *PKR*-R: 5′-ACTACTCCCTGCTTCTGACG-3′; *MX1*-F: 5′-GCACACACCCAACTGTCAGCGA-3′, *MX1*-R: 5′-CCCATGTCCGAAACTCTCTGCGG-3′. *XBP1u*-F: 5′-CCTTGTAGTTGAGAACCAGG-3′, *XBP1u*-R: 5′-GGGGCTTGGTATATATGTGG-3′; *XBP1s*-F: 5′-GGTCTGCTGAGTCCGCAGCAGG-3′, *XBP1s*-R: 5′-GGGCTTGGTATATATGTGG-3′. Human *GAPDH* was measured as an internal control.

### Western Blotting

Cells were harvested and treated with lysis buffer [150 mM NaCl, 50 mM Tris-HCl (pH 7.5), 1 mM EDTA, 1 Mm PMSF, 1% Triton X-100, 1% protease inhibitor cocktails, and 0.5% NP-40]. Western blotting was then performed as previously described (Wang et al., 2019). The anti-GAPDH antibody (Proteintech), anti-ZIKV E protein antibody (BioFront), anti-ERK1/2 antibody (ABclonal), and anti-phospho-ERK1/2 (ABclonal) were used as primary antibodies. Immunoreactive bands were analyzed with an Odyssey infrared imaging system (LI-COR). Quantity One program (Bio-rad) was used to quantify the western blotting results.

### Generation of HCAR2-Knockout Cell Clones

CRISPR/Cas9 system was utilized to generate HCAR2-knockout cell clones as previously described (Ran et al., 2013; Gao et al., 2019). sgRNAs were designed and cloned into the vector, lentiCRIPSR v2 (Addgene #52961). The sequences of sgRNA were shown as follows: HCAR2-sg-F: 5′-CACCGGCCTCACATAGTTGTCCATC-3′, HCAR2-sg-R: 5′-AAACGATGGACAACTATGTGAGGCC-3′. Then, the cell clones were confirmed by genomic DNA extraction and sequencing. The following primers were used for DNA sequencing: HCAR2-sequence-F: 5′-ACCACACAGACACACACCTCCT-3′, HCAR2-sequence-R: 5′-GGGAAAGGATGGGCTGGAGAAGTAGTAC-3′.

### Generation of XBP1-Knockdown Cell Clones

The vector pLKO.1-TRC obtained from Addgene (plasmid # 10878) was used to construct *XBP1*-shRNA or scrambled control (Scr)-shRNA to generate XBP1-knockdown cells as previous described (Chen et al., 2017). Forward oligo of *XBP1*-shRNA1: 5′-CCGGGACCCAGTCATGTTCTTCAAACTCGAGTTTGAAGAACATGACTGGGTCTTTTTG-3′; reverse oligo of *XBP1*-shRNA1: 5′- AATTCAAAAAGACCCAGTCATGTTCTTCAAACTCGAGTTTGAAGAACATGACTGGGTC-3′; forward oligo of *XBP1*-shRNA2: 5′- CCGGAACAGCAAGTGGTAGATTTAGCTCGAGCTAAATCTACCACTTGCTGTTTTTTTG-3′; reverse oligo of *XBP1*-shRNA2: 5′- AATTCAAAAAAACAGCAAGTGGTAGATTTAGCTCGAGCTAAATCTACCACTTGCTGTT-3′. The pLKO.1-*XBP1*-shRNA or pLKO.1-Scr-shRNA was co-transfected with psPAX2 and pCMV-VSV-G into 293T cells. The supernatants were harvested at 48 h post-transfection and filtered with 0.45 μm filters (Millipore). Then, A549 cells were infected with *XBP1*-specific-shRNA-expressing or Scr-shRNA-expressing lentivirus, respectively for 1 h and cultured with fresh medium. Virus-infected A549 cells were selected at 24 h p.i. by puromycin (1 μg/ml) for 1 week and subjected to following experiments.

### Generation of XBP1-FLAG-Expressing Plasmid

Human *XBP1* with a FLAG epitope tag sequence at its 3′ terminus was amplified through PCR with cDNAs of A549 cells as template. The tagged *XBP1* fragment was then inserted into the vector CSII-EF-MCS-IRES2-Venus. Accuracy of CSII-EF-MCS-IRES2-Venus-XBP1-FLAG was confirmed by DNA sequencing.

### Flow Cytometry Analysis

The HCAR2-KO and control cells were infected with ZIKV at an MOI of 1. At 24 h p.i., the cells were suspended in PBS buffer and incubated with anti-ZIKV E antibody (BioFront), followed by incubation with Alexa Fluor 647-conjugated anti-mouse IgG (H+L) Cross-Adsorbed secondary antibody (Invitrogen). Then, labeled cells were examined by flow cytometry (Beckman Coulter, CytoFLEX S).

### Viral Entry Assay

In the viral attachment assay, cells were incubated with ZIKV at an MOI of 3 at 4°C for 1 h. The supernatants of cells were discarded, followed by washing with PBS buffer for three times. In the endocytosis assay, cells were incubated with viral particles at an MOI of 3 at 37°C for 30 min to allow for viral attachment and internalization. The supernatants of cells were then discarded, followed by washing with glycine-HCl pH3.0 buffer for three times. Total RNAs were extracted with Trizol reagent (Invitrogen) and viral RNA levels were detected by qRT-PCR assay.

### Statistical Analysis

All the data were shown as means ± standard deviations (S.D.) from at least three independent experiments. The statistical analysis was performed with an unpaired, two-tailed Student's *t*-test.

## Data Availability Statement

The datasets generated for this study can be found in the NCBI GEO database (https://www.ncbi.nlm.nih.gov/geo/query/acc.cgi?acc=GSE124094), the accession number is GSE124094.

## Author Contributions

CL and PZ designed this project. XM performed most of the experiments. XM, CL, and PZ analyzed the results and wrote the paper. XL, SZ, YH, CC, CH, and LS participated in some experiments.

### Conflict of Interest

The authors declare that the research was conducted in the absence of any commercial or financial relationships that could be construed as a potential conflict of interest.
